# Subsequent attendance in a breast cancer screening program after a false-positive result in the Local Health Authority of Bologna (Italy)

**DOI:** 10.1038/s41598-021-87864-x

**Published:** 2021-04-20

**Authors:** Lorena Squillace, Lorenzo Pizzi, Flavia Rallo, Carmen Bazzani, Gianni Saguatti, Francesca Mezzetti

**Affiliations:** 1 Department of Public Health, LHA Bologna, Via Boldrini, 12, 40121 Bologna, Italy; 2 Department of Biomedical and Neuromotor Sciences, School of Hygiene and Preventive Medicine, University of Bologna, Bologna, Italy; 3 Department of Oncology, LHA Bologna, Bologna, Italy

**Keywords:** Cancer, Medical research, Oncology

## Abstract

We conducted a cross-sectional study to assess the likelihood of returning for routine breast cancer screening among women who have experienced a false-positive result (FPR) and to describe the possible individual and organizational factors that could influence subsequent attendance to the screening program. Several information were collected on demographic and clinical characteristics data. Electronic data from 2014 to 2016 related to breast screening program of the Local Health Authority (LHA) of Bologna (Italy) of women between 45 and 74 years old were reviewed. A total of 4847 women experienced an FPR during mammographic screening and were recalled to subsequent round; 80.2% adhered to the screening. Mean age was 54.2 ± 8.4 years old. Women resulted to be less likely to adhere to screening if they were not-Italian (*p* = 0.001), if they lived in the Bologna district (*p* < 0.001), if they had to wait more than 5 days from II level test to end of diagnostic procedures (*p* = 0.001), if the diagnostic tests were performed in a hospital with the less volume of activity and higher recall rate (RR) (*p* < 0.001) and if they had no previous participation to screening tests (*p* < 0.001). Our results are consistent with previous studies, and encourages the implementation and innovation of the organizational characteristics for breast cancer screening. The success of screening programs requires an efficient indicators monitoring strategy to develop and evaluate continuous improvement processes.

## Introduction

Breast cancer represents the most common cancer and the first cause of cancer death among women worldwide^1^. The mortality attributed to breast cancer has strongly decreased in recent decades, since the institution of mammography as a screening test^2,3^. Despite the reduction in breast cancer mortality and morbidity, mammographic screening is also known to be associated with potential negative effects on women who undergo the tests. Part of the potential harm coming from mammographic screening is related to the chance of obtaining false-positive results (FPR). An FPR is an abnormal screening mammogram requiring supplemental imaging or biopsies to eliminate the diagnosis of cancer. FPRs are quite common among women undergoing mammography. Around 5–10% of mammograms each year could result in an FPR, but the cumulative risk for a woman undergoing several rounds of screening could be higher. It has been estimated that over a 10 years period, 50–61% of women undergoing annual mammography will experience an FPR^4,5^. FPR can cause long lasting psychological consequences, such as fear and anxiety, that could re-occur in occasion of future rounds of screening^6–8^. It is known that prior negative experience with cancer screening may influence a patient’s behaviors and attitudes, including the willingness to obtain future screening, but evidence on this topic seems to be conflicting.

Some studies suggest that women with false-positive mammography (FPM) are more likely to return for subsequent screening^9–13^. Some other studies, on the contrary, found lower screening re-attendance following a FPM, delays in subsequent screening and higher risk of late stage at diagnosis^14–21^. Lastly, further studies found no differences in re-screening rates based on screening mammography outcome^22–26^. Secondary literature suggests that the effect could vary across countries and by population. Clinical and socio-demographic characteristics of women can affect future screening attendance, including education, income, frequency of contacts with a physician, ability to take time off work, age, personal or family history of breast cancer^27–30^. Since regular attendance is a main requirement for a successful screening program, all factors that could deter adherence should be known and minimized.

In order to limit the potential damage caused by bad management of the mammography screening program, maximum emphasis must be placed on quality control and quality assurance. In fact, the European guidelines for quality assurance in breast cancer screening and diagnosis include a summary table of key performance indicators that should be regularly monitored^31^.

In order to achieve the expected benefits and minimize negative outcomes, the Italian group for mammography screening (GISMa), together with the National centre for screening monitoring (ONS) created in 2002 by the Italian Ministry of Health, promotes the collection of data on the implementation of the process and impact indicators agreed on a national level, aiming at monitoring and promoting screening programs nationwide^32^. Until now little data is available about factors influencing attendance at the subsequent round of screening after an FPR in our country. The aim of this study is to assess the likelihood of returning for routine breast cancer screening among women who have experienced an FPR and to describe the possible individual and organizational factors that could influence subsequent attendance to the screening program.

## Methods

### Study design and setting

**Figure 1:**
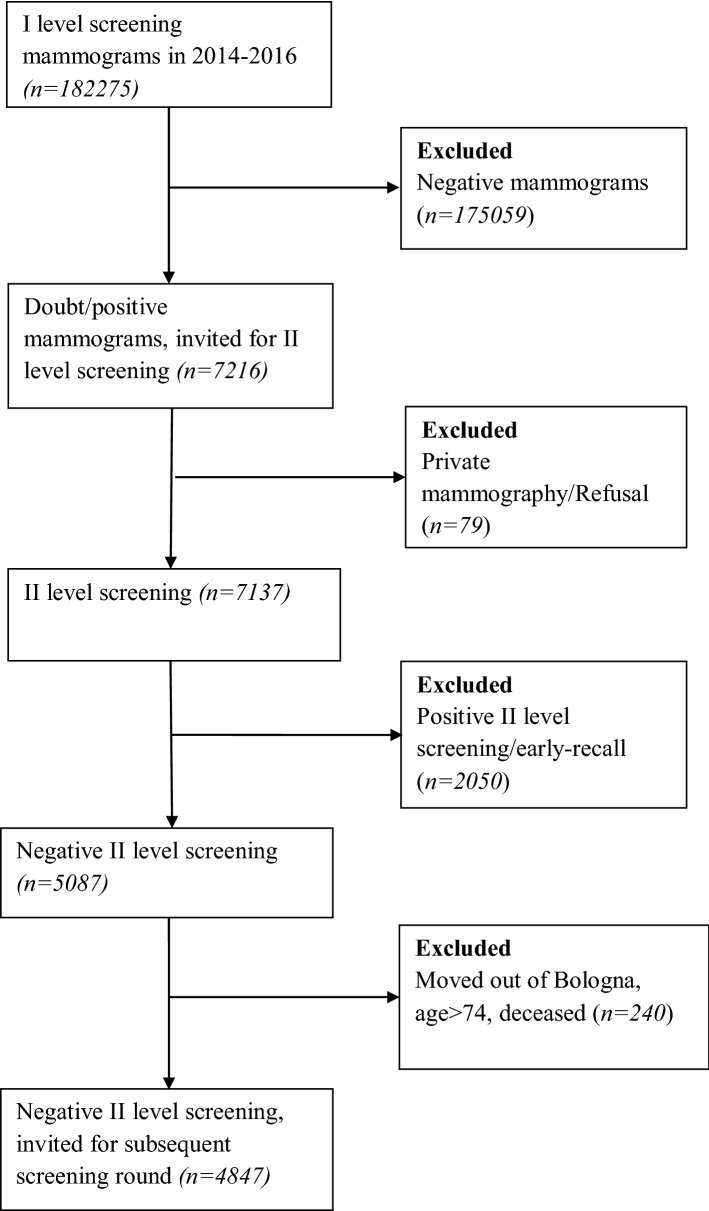
Flow-chart of women’s enrolment.

The study was carried out by retrospectively reviewing electronic data records from 2014 to 2016 related to breast screening program of the Local Health Authority (LHA) of Bologna (Italy). Women between 45 and 74 years old data were obtained; specifically, information included socio-demographic and clinical characteristics. In accordance with previous reports^13–20^, attendance rates at subsequent routine screening rounds were estimated after excluding women who were over the screening age range (i.e., 75 and older), had moved out of LHA of Bologna, had undergone earlier self- or general practitioner-referred private screening, or were deceased. Figure 1 details the steps of our sampling procedure.

### Screening procedure

Breast screening program of the LHA of Bologna routinely screened annually women between 45 and 49 years, and each two years between 50 and 74 years. Eligible women were sent a personal letter inviting them to participate at I level screening test (mammography) at one of the two breast-unit in the area (Hospital 1 with volume of activity in the period 2014–2016 = 160,104 considering I level mammography and Hospital 2 with volume of activity in the period 2014–2016 = 22,171 considering I level mammography). Mammograms were performed by trained radiologist technician and were read independently by two board-certified radiologists with specific training in screening mammography. The evidence suggests that effectiveness of double reading is contingent on whether the two radiologists are blinded to one another's decisions, and how the decisions of the two radiologists are integrated^33^. If the opinions of two radiologists were conflicting, a third reading was requested; each mammogram was categorized as negative if at least 2 radiologists have defined it as negative; otherwise it was considered doubt/positive. If I level conclusion was negative, no further investigations were required; instead, if I level conclusion was doubt/positive, woman was invited to participate at II level screening tests, that provided a clinical breast examination and possible further diagnostic investigations (i.e., breast echography, further mammograms, tomosynthesis, mammotome, cytological and histological examinations). Women were considered to have undergone a false-positive cancer screening test if they had received a doubt/positive result at I level, but negative at the II one. We defined re-attendance in the screening program as participation at the next routine screening round following a screening invitation. Each round begins with the call to the I level mammography and ends with the end of the path ahead related to the invitation of interest.

### Statistical analysis

The independent variables were chosen on the basis of the previous literature^34–38^ and on our hypotheses about the possible association with the dependent variable. Univariate analysis was performed using χ^2^ test for all categorical variables, and Student *t*-test for independent samples to compare all continuous variables. We have adjusted for potential counfounders using multivariable logistic regression model: independent variables for which *p* was 0.25 or less in univariate analysis were included in the multivariate logistic regression models. A two-sided *p*-value of 0.05 or less was considered as indicating a statistically significant difference. The following independent variables were included if they met the above mentioned criteria: age groups (45–49 = 0; 50–69 = 1; 70–74 = 2), nationality (Italian = 0; other = 1), district of residence (Appennino Bolognese = 1; Bologna = 2; Pianura Est = 3; Pianura Ovest = 4; Reno, Lavino, Samoggia = 5; San Lazzaro of Savena = 6), lead time between I level diagnostic mammography and I level diagnostic conclusion (≤ 10 = 1; 11–20 = 2; > 20 = 3), lead time between I level diagnostic conclusion and II level investigations (≤ 5 = 0; > 5 = 1), lead time between II level investigations and II level diagnostic conclusion (≤ 5 = 0; > 5 = 1), Hospital (Hospital 1 = 0; Hospital 2 = 1), invasive investigations (no = 0; yes = 1), previous screening tests (no = 0; yes = 1), age (continuous, in years). The results of the multivariable models are expressed as odds ratio (OR) with 95% confidence interval (95% CI) and *p* values. Statistical analysis was performed by using Stata Statistical Software (Version 14.0)^39^. We confirm that all experiments were performed in accordance with relevant guidelines and regulations.

### Ethical approval

The Ethics Committee of Local Health Authority (LHA) of Bologna (Italy) approved the protocol of the study (Prot.E.C.No. 50-2020-OSS-AUSLBO) in 23/01/2020.

### Consent to participate

Considering the nature of the present study, which was based on reviewing medical records of discharged patients, no written consent was needed by the patients according to the ethics committee Area Vasta Emilia Centro della Regione Emilia-Romagna (CE-AVEC).

## Results

Such as described in Fig. 1, during the study period a total of 7216 women (4% of 182,275 I level screening mammograms in 2014–2016) were invited for subsequent screening round; of these, 4847 experienced an FPR during mammographic screening and were recalled.

Overall, 3885 women (80.2%) adhered to the screening following an FPR. The characteristics of the population are shown in Table 1. Mean age was 54.2 ± 8.4 years old. The women were mostly Italian (92.8%), living in the district of Bologna (60%). Almost three quarters of the women had previous experiences with mammographic screening (72.3%). II level analyses were performed in two different settings: Hospital 1 (67.1%) and Hospital 2 (32.9%). 12% of the women underwent invasive tests, such as needle biopsy and fine needle aspiration. The time period required to complete all screening and diagnostic procedures could vary; for most women the time period from execution of I mammography screening and the availability of screening results was 10 days or less (73%) (lead time 1); lead time from I screening results to execution of II level tests was more than 5 days for 4258 women (88%) (lead time 2), in particular, we have found a significantly longer lead time 2 in Hospital 2 compared to Hospital 1 (94.4% vs 84.9%); lead time from the execution of the II level test to the conclusion of all diagnostic procedures was five or less days for 4189 women (86.6%) (lead time 3).

**Table 1:** Distribution of re-attendance according to several women and diagnostic work-up characteristics.

Characteristics^a^		Re-attendance	
	N (%)	N (%)	*p*
**Total**	4847	3885 (80.2)	
**Age groups (years)**			*p* = 0.683
45–49	2034 (42)	1631 (80.2)	
50–69	2500 (51.6)	2009 (80.4)	
70–74	313 (6.5)	245 (78.3)	
**Nationality**			***p =*** **0.001**
Italian	4498 (92.8)	3630 (80.7)	
Other	348 (7.2)	254 (73)	
**District of residence**			***p <*** **0.001**
Appennino Bolognese	244 (5)	205 (87.2)	
Bologna	2906 (60)	2250 (77.4)	
Pianura Est	693 (14.3)	584 (84.3)	
Pianura Ovest	245 (5)	203 (82.9)	
Reno, Lavino, Samoggia	416 (8.6)	344 (82.7)	
San Lazzaro DI Savena	343 (7.1)	299 (87.2)	
**Lead time 1 (days)**^**b**^			*p* = 0.267
≤ 10	3538 (73)	2823 (79.8)	
11–20	954 (19.7)	782 (82)	
> 20	355 (7.3)	280 (78.9)	
**Lead time 2 (days)**^**c**^			***p =*** **0.004**
≤ 5	580 (12)	491 (84.7)	
> 5	4258 (88)	3385 (79.5)	
**Lead time 3 (days)**^**d**^			***p =*** **0.001**
≤ 5	4189 (86.6)	338 (80.9)	
> 5	649 (13.4)	488 (75.2)	
**Hospitals**			***p <*** **0.001**
Hospital 1	3250 (67.1)	2716 (83.6)	
Hospital 2	1597 (32.9)	1169 (73.2)	
**Invasive investigations**^**e**^			*p* = 0.058
No	4268 (88)	3438 (80.6)	
Yes	579 (12)	447 (77.2)	
**Previous screening tests**			***p <*** **0.001**
No	1344 (27.7)	1022 (76)	
Yes	3503 (72.3)	2863 (81.7)	
**Characteristics**^**a**^	**Mean ± SD**	**N (Mean ± SD)**	**t**
Age (years)	54.2 ± 8.4		*p* = 0.107
Re-attendance		54.3 ± 0.1	
Not re-attendance		53.8 ± 0.27	

Bold values indicates statistically significant difference

^a^Total may not always sum to “n” because of missing data.

^b^Lead time between I level mammography and I level diagnostic conclusion.

^c^Lead time between I level diagnostic conclusion and II level investigations.

^d^Lead time between II level investigations and II level diagnostic conclusion.

^e^Invasive investigations involved cytological and histological examinations.

After the end of II level diagnostic procedures all of the women were invited to participate to the subsequent round of screening with the periodicity required by the protocol of the breast screening program.

Table 1 also shows the results from univariate analyses for factors influencing adherence to subsequent mammographic screening. Women resulted to be more likely to adhere to screening if they were Italian (*χ*^2^ = 12.08; *p* = 0.001), if they not lived in the Bologna district (*χ*^2^ = 36.70; *p* < 0.001), if they had to wait less than 5 days from the result of screening mammography to the execution of the II level test (*χ*^2^ = 8.52; *p* = 0.004), if they had to wait less than 5 days from II level test to end of diagnostic procedures (*χ*^2^ = 11.40; *p* = 0.001), if the diagnostic tests were performed at Hospital 1 (*χ*^2^ = 72.38; *p* < 0.001) and if they had previous participation to screening tests (*χ*^2^ = 19.76; *p* < 0.001). Multivariate stepwise logistic regression analysis results underlined those of the univariate analysis, except for lead time 2 (OR = 0.79; 95% CI 0.62–1.00; *p* = 0.057 ) (Table 2): women resulted to be less likely to adhere to screening if they were not-Italian compared to Italian (OR = 0.73; 95% CI 0.56–0.94; *p* = 0.014), if they lived in the Bologna district compared to who lived in San Lazzaro district (OR = 1.56; 95% CI 1.11–2.20; *p* = 0.011), if they had to wait more than 5 days from II level test to end of diagnostic procedures compared to who had to wait less than 5 days (OR = 0.46; 95% CI 0.29–0.74; *p* = 0.001), if the diagnostic tests were performed at Hospital 2 compared to Hospital 1 (OR = 0.56; 95% CI 0.47–0.67; *p* < 0.001) and if they had no previous participation to screening tests compared to who had did it (OR = 1.51; 95% CI 1.27–1.79; *p* < 0.001).

**Table 2:** Multiple logistic regression analysis results examining re-attendance according to several explanatory variables.

Variable	OR	95% CI	*p*
*χ*^*2*^ = *138.14 (12df), p* < *0.00001, No. of obs* = *4837*			
**Nationality**			
Italian	1.00^a^		
Other	0.73	0.56–0.94	0.014
**District of residence**			
Appennino Bolognese	1.13	0.78–1.63	0.518
Bologna	1.00^a^		
Pianura Est	1.22	0.96–1.56	0.103
Pianura Ovest	1.06	0.74–1.52	0.751
Reno, Lavino, Samoggia	1.05	0.79–1.40	0.725
San Lazzaro of Savena	1.56	1.11–2.20	0.011
**Lead time 2 (days)**^b^			
≤ 5	1.00^a^		
> 5	0.79	0.62–1.00	0.057
**Lead time 3 (days)**^c^			
≤ 5	1.00^a^		
> 5	0.46	0.29–0.74	0.001
**Hospital**			
Hospital 1	1.00^a^		
Hospital 2	0.56	0.47–0.67	< 0.001
**Invasive investigations**^d^			
No	1.00^a^		
Yes	1.48	0.88–2.47	0.138
**Previous screening tests**			
No	1.00^a^		
Yes	1.51	1.27–1.79	< 0.001
**Age (years)**	1.00	0.99–1.01	0.798

^a^Reference category.

^b^Lead time between I level diagnostic conclusions and II level investigations.

^c^Lead time between II level investigations and II level diagnostic conclusion.

^d^Invasive investigations involved cytological and histological examinations.

## Discussion

Since the success of screening programs depends on high adherence rates, our study provides evidence about factors that can reduce adherence of women who have received an FPR. In U.S., women are more likely to re-screen after an FPR (RR 1.07, 95% CI 1.02–12), while in Europe and Canada the odds of future screening are lower (RR 0.97, 95% CI 0.93–1.01; RR 0.63, 95% CI 0.50–0.80, respectively)^40,41^. Consistent with other studies, we have found that 80.2% of women with an FPR returned for a subsequent screening mammogram; this result is in line with 78.3% highlighted by Alamo-Junquera et al. in 2012^34^, and 82% by DeFrank et al. in 2012^42^.

Other factors could influence the return for subsequent screening after an FPR, such as the nature and the extent of supplemental tests needed after mammography. Assessment can include non invasive tests as further mammograms, physical examination, ultrasound, and invasive tests as fine needle aspiration cytology (FNAC) or surgical biopsy. Furthermore, Alamo-Junquera et al. have found that women undergoing biopsy had lower re-attendance to future screenings compared to those who did not (OR = 0.56; 95% CI 0.53–0.59)^34^, whereas in our population it was not a significant difference. Other factors associated with high risk of failing to re-screen have been shown in literature: delays on invitation, longer waiting times in the mammography unit, difficulties in reaching the unit and absence of a formal reminder system^35^.

Our results show a lower adherence in not-Italian women compared to Italian (73% vs 80.7%). This result may depend on difficulties in communication and a poor understanding of the screening program. Some ethnic groups may also have a poor perception of risk due to low literacy levels about breast cancer risk and belonging to hard-to-reach populations^36^. In this view, health education and health literacy programs could have an impact in improving adherence to the screening program in this populations. Multi-lingual health service pathways should be improved, creating a bond between social and health professionals and cultural mediators; primary care team members may also play an important role in providing information and advice^43^.

Women living outside Bologna city area showed more adherence to the screening program than those who living in the city (from 82.7 to 87.2% vs 77.4%). In those contexts, it is much more difficult to turn towards private centers, outside of the Italian healthcare service, in order to reduce waiting times. In addition, the regional healthcare service created points of access using mobile clinics in territories far from the city center where it is much more difficult to find alternatives through private hospitals/centers.

Our findings, at the multivariate analysis, show less adherence in women who had to wait more than 5 days from the second level test to the final diagnostic result, compared to who wait less or equal than 5 days (75.2% vs 80.9%); this time was defined as the “Delay between the assessment and the assessment result” and included in the structural, logistic, organizational and functional indicators according to the GISMa^32^. Moreover, it was considered by the “European guidelines for quality assurance in breast cancer screening and diagnosis” as a cause of anxiety, which sometimes may be considerable; therefore, targets should be set in terms of working days (w.d.) at every stage where delay may arise^33^. Several studies in scientific literature reported negative consequences on screening attendance time in women who received an FPR; women who needed further investigation following a possibly positive or uncertain mammogram may be discouraged from adhering to the screening program^14–21^. Probably, the discomfort already encountered when undergoing a mammography, increased by an FPR, may result in a resilience in going on in further investigations.

Among the indicators and standards for the process evaluation of breast cancer screening, GISMa identifies a desirable “Recall rate (RR)—Further assessment rate” of less than 3% for the baseline test and less than 5% for follow-up tests^32^. The RR is defined as: “the percentage of women who have participated to the mammography screening program who have performed further investigation”. These analyses, which are performed to clarify the nature of an abnormality highlighted by the first level screening mammography can be performed after a woman's recall at a later screening session or at the same level session^32^. Our study shows a greater adherence to the breast screening program in Hospital 1 (RR 4.6%) compared to Hospital 2 (RR 17.8%) (83.6% vs 73.2%). European guidelines recommend that radiologists reporting screening mammograms should read at least 5,000 cases per year. Our results show longer wait time for second test at Hospital 2 (*p* < 0.001), evidencing that the volume of activity can influence the accuracy of cancer diagnosis and the efficiency of screening, as repeatedly observed in other European programs^37,38^. It has been shown that the volume of procedures or patients is a key determinant of quality and efficiency^44^. This indicator should be periodically monitored because a recall with a subsequent benignity outcome represents a negative effect of the screening program and results into additional costs. In addition, women who have experienced an FPRs are unnecessarily worried and some may feel distress which affects their ability to do their normal day-to-day activities. This personal feeling can have a negative impact also on the adherence for the subsequent screening rounds^18^. Hence, the optimal ratio between detection rate and RR must be improved, especially in case of FPR.

Our study has some limitations. The "lead time 2" may also be influenced by the woman's availability to carry out the diagnostic investigations. We have not studied the "delays on invitation" which could affect the adherence, possibly because women could enter into opportunistic screening, as shown in previous studies^35^. Moreover, the Bologna LHA does not have a “formal reminder system" which may reduce the probability that the woman will not perform the booked diagnostic examination.

As highlighted in other similar studies, our data are aggregated and anonymized; thus, we do not have access to detailed demographic risk factors beyond age. As a result, we cannot perform subgroup analyses on variables that are known to affect attendance^45^. Finally, we have not considered/excluded women who have developed cancer in the call range after the FPR.

Despite the mentioned weaknesses, our analysis encourages the implementation and innovation of the organizational characteristics for breast cancer screening; moreover, our study highlights the relevance of adhering to the RR indicator to avoid low participation by women. The success of screening programs requires an efficient indicators monitoring strategy to develop and evaluate continuous improvement processes.

## Data Availability

The datasets used and/or analyzed during the current study are available from the corresponding author on reasonable request.
